# Biologically Active Constituents from *Salix viminalis* Bio-Oil and Their Protective Activity Against Hydrogen Peroxide-Induced Oxidative Stress in Chinese Hamster Ovary Cells

**DOI:** 10.1007/s12010-014-1171-0

**Published:** 2014-08-30

**Authors:** Anna Ilnicka, Katarzyna Roszek, Andrzej Olejniczak, Michal Komoszynski, Jerzy P. Lukaszewicz

**Affiliations:** 1 Faculty of Chemistry, Nicolaus Copernicus University, ul. Gagarina 7, 87-100 Torun, Poland; 2 Faculty of Biology and Environment Protection, Nicolaus Copernicus University, ul. Gagarina 7, 87-100 Torun, Poland; 3 Flerov Laboratory of Nuclear Reactions, Joint Institute for Nuclear Research, Joliot-Curie 6, Dubna, Russia 141980

**Keywords:** *Salix viminalis*, Pyrolysis, Bio-oil, Phenolic extract, Antioxidants, Oxidative stress

## Abstract

The protective antioxidative effect of the phenolic extract (PE) isolated from *Salix viminalis* pyrolysis derived bio-oil was shown in vitro on the Chinese hamster ovary (CHO) cells exposed to hydrogen peroxide (H_2_O_2_). Cells pretreated with 0.05 μg/ml PE after exposure to different concentrations of H_2_O_2_ (300–900 μM) showed up to 25 % higher viability than the unpretreated ones. The antioxidative effect of PE was also observed in a time-dependent manner. The results were confirmed by visual examination of the specimens using microscopy. Finally, superoxide dismutase (SOD) activity modulation was shown by SOD assay, designed to determine the activity of enzymes removing free radicals.

## Introduction

Pyrolysis of biomass is listed among the most promising technologies able to overcome the global challenges arising from a shortage of fossil fuels, increasing demands for energy, and environmental concerns as well. Bio-oil, being the main product of the biomass pyrolysis, has the potential to be used for producing fuels and value-added chemicals.

It is known that bio-oils contain a huge variety of phenolic species resulting from breakdown of lignin, which are responsible for their antioxidant properties. Basically, they can be classified as water-insoluble, high-molecular weight components (pyrolytic lignin) and water-soluble, low molecular weight ones (mainly monomers). The antioxidant properties of whole bio-oils and their fractions consisting of low- and high molecular weight components were proved using various chemical assays [1]. Significant effort has been made for the antioxidant characterization of pyroligneous acids (also known as liquid smoke) obtained by condensation of smoke generated during wood carbonization [2–4]. These liquids have a composition closely similar to that of bio-oils, except for the absence of low-volatile components, like pyrolytic lignin and sugars. Their antioxidant capacity was determined by several methods, including activities against of ABTS radical cation [4], hydroxyl radical (^∙^OH) [5], superoxide radical anion (O_2_^−∙^) [5], and DPPH radical [3–5] as well as phosphomolibdenium [3, 4] and FRAP [3–5] reducing assays. The antioxidant capacity of pyrolysis liquids was demonstrated to be lower than pyrolytic lignin [1, 6], however, still comparable or even superior to that of many synthetic antioxidants, including BHA, BHT, and α-tocopherol [3, 7].

The biological activity has been studied for some of the individual bio-oil components only. Among these studies, one should mention the evaluation of antioxidant, anti-inflammatory, and hepatoprotective properties of vanillin in carbon tetrachloride-treated rats [8], suppression of oxidative stress, and carcinogenesis in *Helicobacter pylori* by 4-vinyl-2,6-dimethoxyphenol treatment [9].

A very limited number of studies have been devoted to investigating the interaction of bio-oils and other wood pyrolysis liquids with biological systems in vivo and in vitro [10, 11]. According to Pimenta et al. [12], eucalyptus-derived pyrolysis liquid and its phenolic fraction are characterized by moderate toxicity, being able to immobilize 50 % of the *Daphnia magna* population after 24-h exposure at concentrations of 170 and 68 mg/l, respectively. No genotoxic effect of the pyrolysis liquid was found, as evaluated by Microtox assay applied with and without exogenous metabolic activation. Similarly, no genotoxic effect of phenolic fraction (lower phase) obtained by fast pyrolysis of Radiata Pine was found, as assessed by Park et al. [13] using the comet assay. The authors observed that the cytotoxicity of the phenolic fraction is much lower than its acute toxicity on *D. magna* and linked this observation to the particular sensitivity of crustacea to methoxy phenols. The upper (nonphenolic) fraction of bio-oil was suggested to contain the compounds of higher cyto- and genotoxicity, but less toxic towards *D. magna*. Chatterjee et al. [14] studied the activity of whole bio-oils produced by slow pyrolysis of rice straw and sawdust. They demonstrated that cytotoxicity, apoptosis, and genotoxicity in Jurkat T (human lymphoma) and HepG2 cell cultures increases with the bio-oil dose. Similarly, a dose-dependent relation was observed for in vivo survivability of *Caenorhabditis elegans*. Recently, a beneficial effect of hickory shell-derived pyroligneous acid was demonstrated on *C. elegans* exposed to oxidative stress in vivo [15]. Hong et al. [16] studied pyrolyzates derived from low-temperature heat treatment (150–200 °C) of bamboo. Treatment with high concentrations of pyrolyzates leads to the decreased cell viability, whereas cells treated with low concentrations showed higher viability than control. The protective effect of the pyrolyzates against *N*-methyl-d-aspartate (NMDA)-induced apoptotic cell damage in cortical neurons was demonstrated as well.

In our previous studies, we demonstrated the successful isolation of phenolic constituents from *S. viminalis* pyrolysis bio-oil. Chromatographic analysis revealed that the high ratio of syringylpropane units to guaiacylpropane units characteristic of the lignin of *S. viminalis* leads to high levels of syringol and its para-substituted derivatives in the bio-oil. The results of quantitative gas chromatographic analysis of the phenolic extract are presented in [17]. The ingredients work is known to exhibit high antioxidative efficiency resulting from the electronic effects of ortho-methoxy and para-substituents. So far, we examined phenolic components of *S. viminalis*-derived pyrolysis oil with regard to their potential application to the oxidative stabilization of synthetic lubricating oils. In these studies, two phenolic fractions were isolated through solvent extraction (extract A was ether soluble; extract B was methylene chloride soluble) and characterized by chromatographic analysis. Both extracts showed bang up the antioxidative effect of the obtained extracts on PAOs and diesters, high-temperature oxidation tests. Nevertheless, there are no reports in the literature concerning the bio-oil from *S. viminalis* and its antioxidant activity against oxidative stress-treated cells in vivo which prompted us to undertake this type of research.

A variety of pathological states have been associated with oxidative damage or stress that is mediated by excessive exposure of cells to reactive oxygen species such as free radicals [18]. Hydrogen peroxide can be the source of one of the most reactive free radicals—hydroxyl radical. Prime targets for free radical reactions are the unsaturated bonds in membrane lipids. Consequent peroxidation results in a loss in membrane fluidity and potentially in cellular lysis. Other effects of free radical damage include DNA mutations and inactivation of disulfide bounds containing enzymes [19–22].

## Materials and Methods

### Separation of Phenolic Antioxidants from Bio-Oil

Bio-oil was obtained in the process of thermal treatment of *S. viminalis* wood, according to the procedure described in [17]. The fractions of phenolic antioxidants were isolated from the resulting bio-oil by a two-step solvent extraction procedure using the Scholze and Meier method [23]. Briefly, the designated amount of bio-oil was added dropwise and under vigorous stirring into chilled deionized water. Next, the water-insoluble pyrolytic lignin fraction was removed by vacuum filtration. The water-soluble phase was extracted with diethyl ether and then with methylene chloride. Finally, the organic solvents from the resulting fractions were removed in a rotary evaporator under the reduced pressure. As it was demonstrated earlier, the ether-soluble fraction contained higher concentration of phenolic antioxidants and performed significantly better in the high-temperature oxidation tests. Thus, in the present study, only this fraction labeled as PE was assayed for protective action against H_2_O_2_-induced oxidative stress in vitro. Detailed composition of the low-molecular-weight components of the ether-soluble fraction is reported in [17].

### Cell Cultures

CHO cell line was obtained from Sigma-Aldrich. The cells were grown in Ham’s F-12 medium containing 10 % FBS, 100 U/ml of penicillin, and 100 μg/ml of streptomycin in a humidified atmosphere of 5 % CO_2_ at 37 °C for 24 h prior to the experiment.

### Assessment of the Cytotoxicity of PE Towards CHO Cells

The ether extract showed a higher antioxidant activity. Therefore, the same extract was used in the next stage of the study on the biological activity. The viability of the cells was assessed by the MTT assay. The yellow MTT [3-(4,5-dimethylthiazol-2-yl)-2,5-diphenyltetrazolium bromide] is reduced to a purple formazan by mitochondrial enzymes. To determine the highest concentration of PE which does not induce any apparent cytotoxicity, the CHO cells were incubated for 24 h with different concentrations of PE. The cytotoxicity was basically assessed the MTT assay according to [24, 25] after preliminary examination by Trypan blue exclusion test [26]. Based on these preliminary tests, the PE concentration of 0.05 μg/ml was chosen for antioxidative assays in vitro.

### Protective Effect of PE Against H_2_O_2_-Induced Oxidative Stress in Vitro

CHO cells were subjected to two different treatment regimens involving H_2_O_2_ as oxidative stress inducing agent. In the first experiment, both the unpretreated cells and the cells pretreated for 24 h with 0.05 μg/ml PE were exposed to different concentrations of H_2_O_2_ (300 to 900 μM) for the next 24 h. Second experiment was carried on analogously, except that the cells were exposed to H_2_O_2_ at the highest concentration of 900 μM and the treatment time was 12, 24, or 48 h. The viability of CHO cells in both experiments was determined by the MTT assay and expressed as the percent ratio of the control sample.

### SOD Activity

SOD activity was determined by the method of Beauchamp and Fridovich based on the inhibition of the nitroblue tetrazolium (NBT) reduction reaction [27]. The product of NTB reduction was detected spectrophotometrically at 560 nm. The final SOD activity was expressed in SOD units per milligram of the protein in the sample, where SOD units express the amount of the SOD enzyme required to inhibit the reduction of NBT by 50 %.

### Determination of Protein Content—Bradford Assay

The Bradford assay is a protein determination method that involves the binding of Coomassie Brilliant Blue G-250 dye to proteins [28]. The dye exists in three forms: cationic (red), neutral (green), and anionic (blue). Under acidic conditions, the dye is predominantly in the doubly protonated red cationic form (Abs max = 470 nm). However, when the dye binds to protein, it is converted to a stable unprotonated blue form (Abs max = 595 nm). It is this blue protein-dye form that is detected at 595 nm in the assay using a spectrophotometer.

The CHO cells were lysed using cell lysis buffer (Cell Signaling Technology), containing 1 % Triton X-100 and 1 mM PMSF (proteases inhibitor), according to the manufacturer’s protocol. The cell lysate diluted in Tris–HCl buffer, pH = 7.6 was mixed with a Bradford reagent. After 5 min, the absorbance at 595 mm was recorded.

## Results and Discussion

To determine the range of effective concentrations of hydrogen peroxide, capable to induce oxidative stress, CHO cells cultured for 24 h without addition of PE were treated for the next 24 h with increasing concentrations of H_2_O_2_. The viability results are summarized in Table 1.

**Table 1:** The viability of CHO cells treated with increasing concentrations of hydrogen peroxide

Sample		Viability (%)
Cells without pretreatment	Control^a^	100
	300 μM H_2_O_2_	54
	600 μM H_2_O_2_	43
	900 μM H_2_O_2_	40
Cells pretreated with 0.05 μg/ml PE for 24 h	Control^a^	100
	300 μM H_2_O_2_	79
	600 μM H_2_O_2_	65
	900 μM H_2_O_2_	51

The viability of cells was determined by the MTT assay

^a^The control sample –CHO cells cultured in standard conditions without hydrogen peroxide addition

H_2_O_2_ in the concentrations ranging from 300 to 900 μM decreased the proliferation and viability of CHO cells to 54, 43, and 40 % of the control, respectively. On the other hand, the decrease in viability of cells pretreated with PE was lower by 25–11 %, depending on the H_2_O_2_ concentration.

**Fig. 1:**
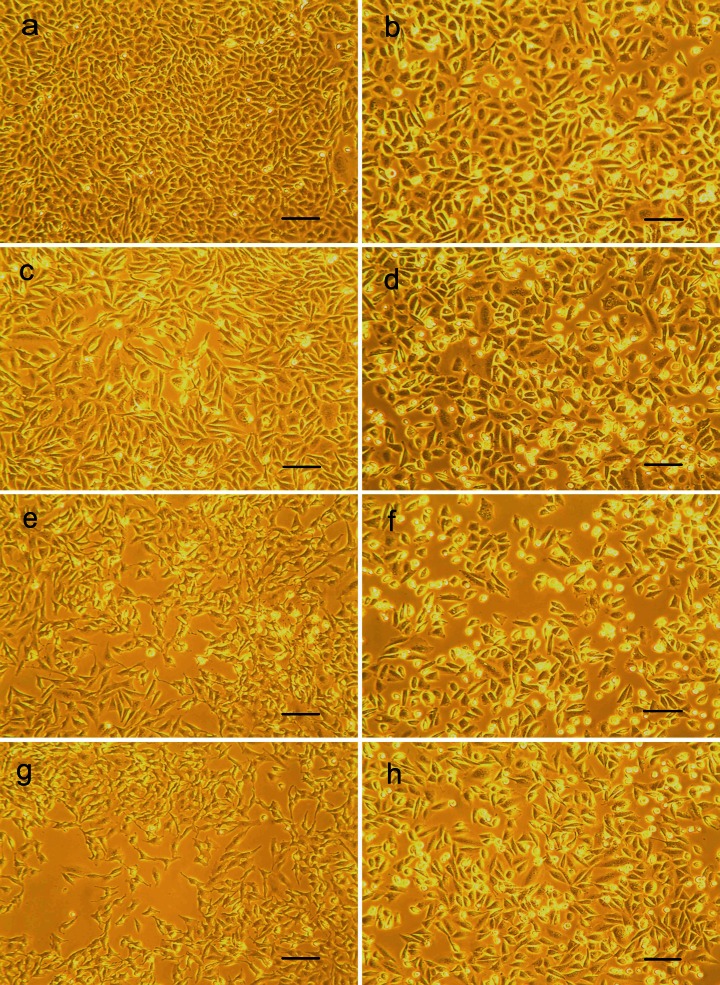
Chinese hamster ovary (CHO) cells 72 h after passaging: cultured without any supplementation (**a**), pretreated with 0.05 μg/ml phenolic extract for 24 h, without hydrogen peroxide addition (**b**), cells exposed for 24 h to 300 μM H_2_O_2_ (**c**), 600 μM H_2_O_2_ (**e**), and 900 μM H_2_O_2_ (**g**), cells pretreated with 0.05 μg/ml phenolic extract for 24 h and then exposed for 24 h to 300 μM H_2_O_2_ (**d**), 600 μM H_2_O_2_, (**f**) and 900 μM H_2_O_2_ (**h**). *Scale bar* 100 μm

**Fig. 2:**
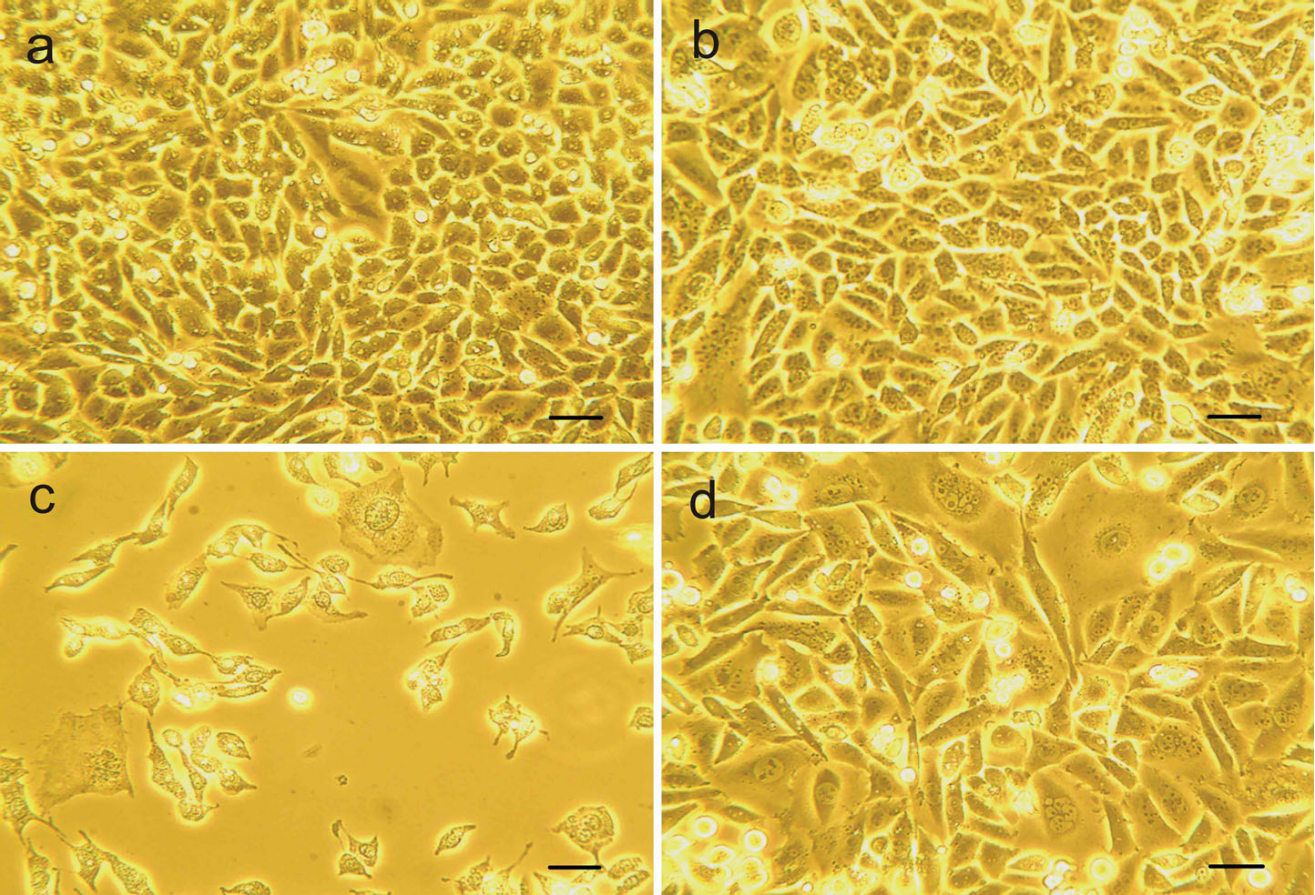
Chinese hamster ovary (CHO) cells 72 h after passaging: cultured without any supplementation (**a**), pretreated with 0.05 μg/ml phenolic extract for 24 h, without hydrogen peroxide addition (**b**), cells exposed for 48 h to 900 μM H_2_O_2_ (**c**), cells pretreated with 0.05 μg/ml phenolic extract for 24 h and then exposed for 48 h to 900 μM H_2_O_2_ (**d**). *Scale bar* 50 μm

On the microscopic observations, the morphological changes triggered probably by intracellular ultrastructural damages were visible by the treatment with 300 to 900 μM H_2_O_2_ (Fig. 1c, e, g). We found that pretreatment with 0.05 μg/ml phenolic extract not only increases the viability of CHO cells exposed to various concentrations of hydrogen peroxide, but also maintains unchanged morphology (Fig. 1d, f, h). The antioxidative effect of pretreatment with 0.05 μg/ml phenolic extract was also observed with CHO cells exposed to 900 μM H_2_O_2_ in time-dependent manner (Table 2; Fig. 2). After 48 h of culture in the presence of 900 μM H_2_O_2_, less than 1 % of CHO cells remained alive. Whereas cells pretreated with PE showed lower reduction in their viability and after 48-h treatment, still 24 % of cells remained alive.

**Table 2:** The viability of CHO cells treated with 900 μM hydrogen peroxide for 12, 24, and 48 h

Sample	Viability of cells without pretreatment (%)	Viability of cells pretreated with 0.05 μg/ml PE for 24 h (%)
Control^a^	100	100
12 h	54	62
24 h	36	42
48 h	<1	24

The viability of cells was determined by the MTT assay

^a^The control sample –CHO cells cultured in standard conditions without hydrogen peroxide addition

We have also determined the activity of superoxide dismutase as one of the possible mechanisms of cell defense against free radicals. The SOD activity was raised in the cells pretreated with the phenolic extract (Table 3).

**Table 3:** SOD activity determination of CHO cells treated with 900 μM hydrogen peroxide for 24 h

Sample		SOD activity (U/mg protein)
Cells without pretreatment	Control^a^	0.230
	900 μM H_2_O_2_	0.280
Cells pretreated with 0.05 μg/ml PE for 24 h	Control^a^	0.335
	900 μM H_2_O_2_	0.323

The SOD activity of cells was determined as described in “Materials and Methods” section

^a^The control sample –CHO cells cultured in standard conditions without hydrogen peroxide addition

The process of slow pyrolysis of *S. viminalis* wood results in generation of charcoal, bio-oil, and biogas [29]. The phenolic fractions extracted from the bio-oil of *S. viminalis* contain a range of compounds with antioxidative properties. Proven antioxidant activity of *S. viminalis* bio-oil-derived PEs, especially reinforced in hydrogen-bond-accepting (HBA) solvents, suggests their potential applicability for the antioxidant protection.

To test its protective biological activity, we added various concentrations of PE to CHO cell line cultured in standard conditions. In preliminary experiments, we determined the maximum dose of PE that was nontoxic to the CHO cells (data not shown). We have found that virtually no difference was seen in cell number and viability between controls and cells treated with 0.05 μg/ml PE.

We have tested the viability of CHO cells pretreated for 24 h with 0.05 μg/ml extract and exposed for various concentrations of hydrogen peroxide for the next 24 h. Our results indicate that exposure of CHO cells to various concentrations of H_2_O_2_ resulted in decreasing of cells viability expressed by activity towards MTT. We have also observed that CHO cells exposed to high doses of H_2_O_2_ changed their morphology—the cells were smaller and shrunken in comparison with unaffected cells. This was probably caused by oxidative damage of cell membrane and changes in its permeability. After exposure to H_2_O_2_, cells pretreated with 0.05 μg/ml PE not only showed 11 to 25 % higher viability than the unpretreated ones, but also maintained unchanged morphology. The antioxidative effect of pretreatment with 0.05 μg/ml phenolic extract for 24 h was also observed with CHO cells exposed to 900 μM hydrogen peroxide in time-dependent manner. In this case, the exposition time of 48 h was long enough to induce cell death in all CHO cells, whereas PE pretreated cells still showed the viability of 24 %.

It has to be stated that the phenolic extract was used without any extensive purification and as such may contain compounds of proven cytotoxic activity, e.g., phenol, catechol, etc. Phenol induces diverse effects on the epithelial barrier function in vitro. These cellular membrane microdomains are affected by phenol exposure, thus destabilizing the tight junction proteins that regulate epithelial barrier function in cells, as described in [30]. Phenol also elicits a strong cytotoxic effect when added to PK-15 cells [31]. Oliveira et al. studied cytotoxicity of catechol [32]. Catechol induced time- and concentration-dependent cytotoxic effects. Morphological changes, such as the retraction of the cytoplasm and chromatin clumping, were seen in cells exposed to catechol for 48 h. Thus, the obtained result should be understood in terms of trade-off between the harmful action of these compounds and protective activity due to the presence of components with high antioxidative potential. Based on the previously reported results showing that PE from *S. viminalis* bio-oil are composed mainly of 2,6-dimethoxyphenol (syringol) and its para-substituted derivatives, the high antioxidative activity could be explained by the electronic effects of ortho-methoxy and para substituents. 2-Methoxyphenol (guaiacol) and its derivatives, exhibiting low antioxidant activity, are present at much lower concentrations. Even lower is the content of potentially harmful components resulting from the decomposition of phenylpropane lignin units (i.e., phenol and its p-substituted derivatives). One should note that the share of the identified low-molecular-weight components accounted only for 50 wt%, (GC based). Our mass spectrometric studies revealed the presence of lignin dimers with molecular weight ranging from 250 to 400 amu. Some studies suggest that these are highly valuable components with lignane-like structure; however, the exact structure of lignin dimers originating from *S. viminalis* is so far unsolved.

The positive results of antioxidative tests encouraged us to undertake an attempt to recognize the possible mechanism of antioxidative activity of polyphenols from phenolic extract of *S. viminalis*. Cellular defense mechanisms against oxidative damage include enzymatic conversion of ROS to less reactive species, chelation of transition metal catalysts, and detoxification of ROS by antioxidants [33]. It is known that antioxidants may modulate the level of endogenous enzymes, such as superoxide dismutase [34]. Superoxide dismutase catalyzes the dismutation of the superoxide anion radical (O_2_^−·^) and as such provides an important defense mechanism against O_2_^−·^-induced toxicity. Our results indicate that SOD activity was slightly raised in the cells exposed to 900 μM H_2_O_2_, while the increase in the SOD activity was observed in CHO cells pretreated with the phenolic extract. The second observation is in agreement with recent results on antioxidant properties of vanillin in vivo, showing the ability of vanillin to increase the activity of hepatic enzyme antioxidants, including catalase and SOD [35]. This is an important factor, as the endogenous enzymes are normally easily deactivated by prolonged oxidative stress. An increased SOD activity in PE-pretreated cells compared to that of controls may indicate, however, that PE induces some level of oxidative stress.

## Conclusions

In conclusion, our results demonstrate that *S. viminalis* bio-oil-derived phenolic extract protects cells against H_2_O_2_-induced oxidative stress damage. Questions on the precise mechanism of phenolic extract action and its effectiveness in various cells in vitro models remain to be answered. In the future, the new objective of our research can be assaying the biological activity of the individual compounds isolated from phenolic extract, being a complex mixture.
